# Tuning the porosity of biofabricated chitosan membranes in microfluidics with co-assembled nanoparticles as templates†
†Electronic supplementary information (ESI) available. See DOI: 10.1039/d0ma00073f


**DOI:** 10.1039/d0ma00073f

**Published:** 2020-03-11

**Authors:** Khanh L. Ly, Christopher B. Raub, Xiaolong Luo

**Affiliations:** a Department of Biomedical Engineering , Catholic University of America , Washington , DC 20064 , USA; b Department of Mechanical Engineering , Catholic University of America , Washington , DC 20064 , USA . Email: luox@cua.edu

## Abstract

Biopolymer membranes assembled in microfluidic devices offer many biological process- and analysis-related applications. One of the key characteristics of bio-fabricated membranes is their porosity, which regulates the transport of molecules, ions, or particles and contributes to their semi-permeability and selectivity. This study aims to tune the porosity of biofabricated chitosan membranes (CM) using incorporated nanoparticles as templates. CM with polystyrene nanoparticles (CM-np) were assembled by flow in microchannel networks. The membranes with incorporated nanoparticles were crosslinked with glutaraldehyde, and then the nanoparticles were dissolved with dimethyl sulfoxide. The *in situ* synthesized porous CM (pCM) were characterized with scanning electron microscopy and polarized light microscopy. Permeability tests confirmed the increased pore sizes of the pCM and enhanced permeability to macromolecules. Sharper static gradients in three-channel microfluidic devices were demonstrated with the pCM as compared to those with the original CM. The capability to customize the porosity of flow-assembled, freestanding and robust biopolymer membranes inside a microfluidic network is attractive and broadens the applications of these membranes in biomolecular and cellular studies.

## Abstract 2

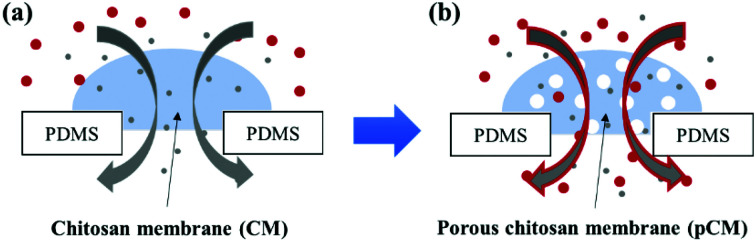
Tuning the membrane porosity in microfluidics with co-assembled nanoparticles as templates for enhanced mass transport and biomacromolecule gradient generation.

## Introduction

The integration of membranes in microfluidics has attracted significant interest over the last two decades to offer precise mass transport for filtration, extraction, and gas–liquid exchange.^1^,^2^ One of the most important attributes to the mass transport of membranes for selective subjects is semi-permeability. The transport is based on a difference in chemical potential between the two sides of a semi-permeable membrane that allows selective subjects to diffuse through.^3^ Since the membrane porosity governs the transport, it is highly desired that the porosity can be actively tuned and customized to enhance the implementation of integrated membranes. Many approaches have been employed to integrate membranes into microfluidics, including direct incorporation of commercially available membranes, membrane preparation as part of the chip fabrication process, *in situ* preparation of membranes, and the direct use of the membrane properties of a bulk chip material.^3^ Among them, the *in situ* biofabrication of chitosan membranes (CM) by locally generated pH gradients has been an attractive method for the integration of biopolymer membranes on-chip.^4^

Chitosan is a partially deacetylated derivative of chitin and commonly used in a variety of biomedical applications.^5^ In particular, chitosan is a well-known pH-responsive biopolymer which is a water-soluble cationic polyelectrolyte at low pH and becomes insoluble with gel or film forming characteristics at higher pH than its p*K*_a_ at 6.3.^6^ Based on this unique property, we were able, in previous studies, to biofabricate *in situ* an insoluble CM, using a flow-assembly method, onto a polyelectrolyte complex membrane (PECM). The PECM was spontaneously formed with electrostatic interactions by bringing a negatively charged alginate solution and a positively charged chitosan solution into contact.^7^ The CM were uniform, robust, and semipermeable to small molecules.^1^ With a recently developed air bubble steering technique, the CM biofabrication process was significantly simplified while precisely maintaining spatial and temporal control of membrane growth.^8^ Furthermore, the resistance of CM to acids and Pluronic was improved with glutaraldehyde crosslinking in a recent report.^9^ However, no study has yet attempted to tune the porosity of this flow-assembled CM in microfluidic networks.

There are several commonly used methods to manipulate membrane porosity including interfacial polymerization, phase inversion, and post-treatment of a porous structure.^10^ In this study, we incorporated polystyrene nanoparticles as a sacrificial template during the *in situ* membrane biofabrication process to tune the membrane porosity. We chose polystyrene as a sacrificial material because it possesses controllable physiochemical properties and has been commonly employed as a template in the synthesis of nanostructured porous and hollow materials with control over the cavity, internal structure and external shape.^11^–^13^ Furthermore, the process to utilize polystyrene as a template is versatile, involving a self-assembly process of the host component with polystyrene to form a composite, followed by a polystyrene removal process.^14^ Importantly, polystyrene is commercially available in a wide range of sizes, giving the ability to actively tune the porosity of the fabricated membranes according to application needs. One such need is to generate static chemical gradients of small molecules, ions or biomacromolecules such as proteins for biological studies.

Chemical gradients play an important role in a variety of biological processes such as directing cellular responses during inflammation, wound healing, chemotaxis, differentiation, and many others.^15^–^18^ Compared to traditional gradient generation platforms where chemical gradients are often ambiguous over space and time and difficult to characterize quantitatively,^16^,^19^ microfluidic-based gradient generators have emerged as promising alternatives that offer time- and cost-savings and yet are highly controllable.^20^–^22^ In our previous study, we developed a static gradient generator composed of parallel and semipermeable CM in a single-layer polymethyl-siloxane (PDMS) microfluidic device. Compared to commercial membranes that are generally sandwiched on the top of serpentine tree-like or Y-shape flow-based gradient generators and required extra effort and care for device packaging,^22^–^24^ our static gradient generator is simple and mild. Furthermore, this static gradient generator can generate quickly responsive and well-maintained gradients of small molecules and pheromones over time, and can be functional and stored for up to one month in moist conditions.^25^ However, the gradient generation of macromolecules using this approach remains a challenge due to the low molecular weight cut-off of CM.

**Fig. 1:**
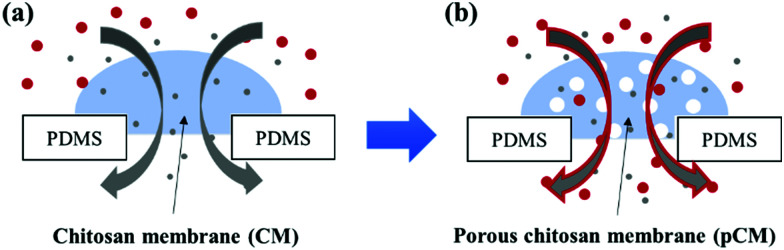
A schematic of transport across membranes. (a) Original CM with a low molecular weight cut-off (∼6 nm); and (b) pCM with tunable porosity using polystyrene particles (25 nm or 200 nm) as a template.

Here, we aim to develop an approach to actively tune the porosity of biofabricated CM (Fig. 1) for broader applications. We directly co-assembled CM with polystyrene nanoparticles (CM-np) in a microchannel network using a flow-assembly method. The CM-np were then crosslinked with glutaraldehyde (GA) to strengthen the membrane's mechanical properties and prevent the collapse of the porous structure during the nanoparticle removal process.^26^ Finally, the nanoparticles were removed using dimethyl sulfoxide (DMSO), resulting in porous CM (pCM). Permeability and static gradients of macromolecules through the pCM were demonstrated and compared with those through the original CM, confirming the capability to actively tune the membrane porosity according to application needs.

## Experimental

### Materials

Sylgard 184 and its curing agent for PDMS device fabrication was purchased from Ellsworth Adhesives. PTFE tubing of 0.022′′ ID and 0.042′′ OD was purchased from Cole-Parmer. Stainless steel catheter plugs and hollow metal couplers of 22-gauge size were purchased from Instech Laboratory Inc. Disposable syringes of 1 mL volume were purchased from Becton, Dickinson and Company. Sodium alginate powder, chitosan flakes (85% deacetylated, medium molecular weight), fluorescein isothiocyanate (FITC, a molecular weight of 389 Da), FITC-labelled dextran (F-dextran) with a molecular weight of 4, 10 and 70 kDa, and GA solution (25% in H_2_O) were obtained from Sigma Aldrich, USA. DMSO solution (ACS grade) was purchased from Amresco® Company. PBS 10× solution of pH 7.4 was purchased from TissuePro Technology, LLC (USA). Polystyrene nanoparticles (1.06 g mL^–1^) were purchased from DegradeX Phosphorex, Inc. All other chemicals can be purchased from major suppliers.

0.5% w/v alginate solution was prepared by dissolving sodium alginate powder in deionized (DI) water and stirring overnight, and the solution was then stored at 4 °C for long-term use (up to 6 months). 0.5% w/v chitosan solution was prepared by dissolving chitosan flakes in DI water with HCl added dropwise to pH 2 and stirring overnight, followed by dropwise addition of 1 M NaOH to adjust the pH to 5–5.5. DI water was added to bring it to the final concentration. The resulting chitosan solution was then filtered with a filter funnel of extra coarse porosity (a pore size of 170–220 μm) and stored at 4 °C. GA solution of 25% was diluted in PBS to obtain the final concentrations of 10% and 4%. FITC and F-dextran of 1 mg mL^–1^ in PBS were prepared for semi-permeability and gradient generation experiments.

### Microfluidic device fabrication

Single aperture and three-channel molds for device fabrication and an add-on vacuum layer were fabricated using conventional photolithographic techniques with negative photoresist SU-8 3035 on 4′′ silicon wafers. The polydimethylsiloxane (PDMS) microfluidic device is fabricated using a conventional soft-lithography method. Briefly, Sylgard 184 and its curing agent were mixed at a 10 : 1 ratio, degassed, poured on top of the molds sitting in an in-house-made aluminum foil container, and cured at 65 °C on a hotplate for 4 hours. The solidified PDMS was then delaminated from the molds and cut into the desired pieces. Microchannels were punched for input and output connections, while the add-on vacuum chamber layer was punched with only one output port. Oxygen plasma (200 mTorr, 10 psi gas source from an oxygen tank, 30 seconds, medium RF level) was used to bind the punched PDMS channels to either cleaned glass slides or flat PDMS bottom layers that can be punched or dissected for morphological observations of the membranes, using Plasma Cleaner PDC-32G (Harrick Plasma). The bonded devices were then put in an oven at 120 °C and left at least overnight to restore the hydrophobicity of PDMS before the membrane biofabrication.

**Fig. 2:**
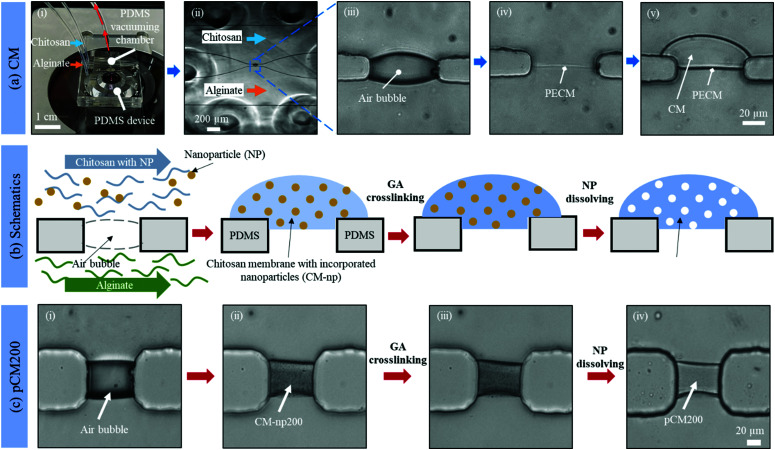
Tuning the porosity of CM with co-assembled nanoparticles as templates. (a) Assembly of the original CM: (i) a PDMS device with an add-on vacuum chamber on a microscope stage; (ii) the microchannels under a vacuum with introduced alginate and chitosan solutions; (iii) an air bubble trapped inside the aperture between microchannels; (iv) the polyelectrolyte complex membrane (PECM) formed after dissipating the air bubble; and (v) growing the CM to the desired thickness under flow at 1 μL min^–1^. (b) Schematics and (c) the corresponding microscopic images showing the tuning process to form the pCM: (i) air bubble trapped inside the aperture between microchannels; (ii) co-assembled CM with polystyrene nanoparticles of 200 nm in diameter (CM-np200); (iii) crosslinked CM-np200 using glutaraldehyde (GA); and (iv) pCM200 after dissolving the polystyrene nanoparticles with DMSO.

**Fig. 3:**
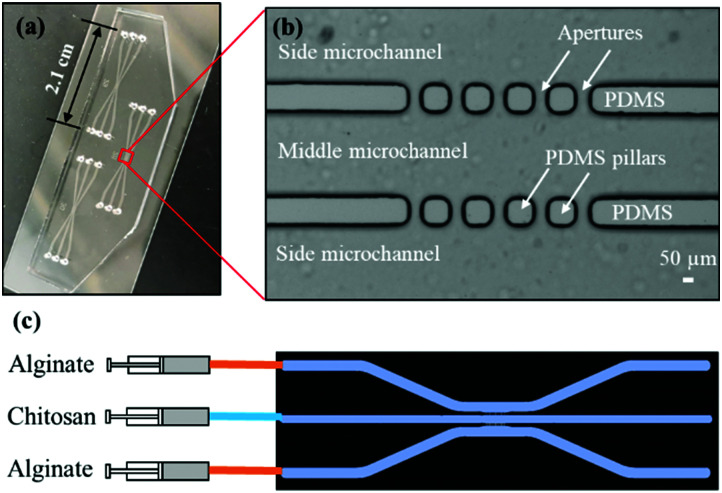
Three-channel microfluidic device and experimental setup to generate static gradients. (a) A device with three sets of PDMS microchannels bonded to a 3′′ × 1′′ glass slide; (b) zoom-in view of the aperture area of one microchannel network; and (c) experimental process to pump alginate and chitosan or chitosan with the nanoparticle solution into the microchannels for the biofabrication of parallel and semipermeable biopolymer membranes.

Fig. 2(a)-(i) and (ii) show a single aperture device used for membrane characterization in this study. This device consisted of two channels with two inputs and two outputs, and each microchannel is 500 μm × 40 μm in width and height, correspondingly, except around the aperture region where the two microchannels gradually widen as shown in Fig. 2(a)-(ii). Fig. 3(a) and (b) show a microfluidic network comprising three channels. The dimension of the entire microchannel networks was 50 μm in depth. The microchannels were 500 μm wide in the tubing connection section and smoothly curved and gradually narrowed to 220 μm wide near the aperture area where the biopolymer membranes were fabricated as reported in previous studies.^4^,^8^,^25^ The ten apertures and eight PDMS pillars between the apertures were 50 μm in all three dimensions.

### Chitosan membrane (CM) biofabrication

Basic alginate solution (pH 11.5) was adjusted with 1 M NaOH solution dropwise before use. Alginate and chitosan (pH 5.5) solutions were introduced at a flow rate of 1 μL min^–1^ separately into the two side microchannels of the single-aperture device, and the outputs were left open (Fig. 2(a)-(i and ii)). In the case of the three-channel device, chitosan solution was introduced into the middle channel, while alginate solutions were introduced into the two side microchannels (Fig. 3(c)). When the solutions came to the aperture (or apertures in the three-channel devices), an air bubble (or air bubbles) was trapped inside the aperture(s) due to the hydrophobicity of the PDMS surfaces, and the pumps were stopped for the flows to settle. Then we followed the reported steering air bubble technique to remove the air bubble(s).^8^ In particular, the add-on vacuum layer was connected by PTFE tubing to a 3 mL syringe on a withdrawal syringe pump. The surface of the add-on vacuum layer was gently cleaned with isopropanol, placed upon the aperture area, and gently pressed to ensure adherence (Fig. 2(a)-(i)). Withdrawing about 1.5 mL of the connected syringe initiated the vacuum process and began to shrink the air bubble(s) in about 5 min (Fig. 2(a)-(iii)). After complete dissipation of the air bubble(s), a thin PECM instantaneously formed in the aperture(s) upon contact between the positively charged chitosan and negatively charged alginate macromolecules (Fig. 2(a)-(iv)). Once the PECM was formed, the flows were restarted, allowing CM to grow upon the PECM *via* the diffusion of hydroxyl ions from the alginate side through the PECM to the chitosan side. Within 2–3 minutes, the desired CM thickness of about 30 μm was achieved (Fig. 2(a)-(v)). Next, the add-on vacuum layer was removed, the flows were stopped, and the tubing was disconnected. Finally, the microchannels were manually rinsed with PBS. The membranes were robust enough to withstand manual rinsing.

### Porous chitosan membrane (pCM) biofabrication

Fig. 2(b) shows schematics of the fabrication process to tune the membrane porosity. Chitosan solution mixed with polystyrene nanoparticles (25 or 200 nm in diameter) was prepared by mixing chitosan (0.5% w/v) and polystyrene (1.06 g mL^–1^) solutions at a volume ratio of 20 : 1. Next, CM-np were assembled by flows in the same manner as the CM. Then the CM-np were crosslinked with 10% GA by introducing GA solution into the microchannels to soak the CM-np with GA molecules and left at room temperature for one hour. After that, all channels were manually rinsed thoroughly with PBS. Next, DMSO solution was introduced into the microchannels and left at room temperature for two hours to dissolve the polystyrene. The fabrication process was monitored under a Ludesco EXI-310 inverted microscope (Fig. 2(c)). Eventually, the channels were manually rinsed with PBS and the fabricated pCM from the 25 or 200 nm nanoparticle template (pCM25 or pCM200, correspondingly) were stored at 4 °C for further analysis. The membranes were robust enough to withstand manual rinsing.

### Membrane morphology observation

For morphology observation purposes, the single aperture devices were made of two PDMS layers. After membrane fabrication, the resulting CM, CM-np, and pCM were washed with PBS, then fixed in 4% GA for 20 minutes, and rinsed thoroughly with PBS twice before being dehydrated with an ascending series of isopropyl (50%, 60%, 70%, 80%, 90%, 95%, and 100%). Once dried, the samples were taken out of the two-layer PDMS devices by punching using a biopsy punch (2 mm diameter). The two PDMS layers of the punched samples were gently delaminated, and the membranes on one of the PDMS layers were confirmed under a light microscope. For nanoscale morphology observation by scanning electron microscopy (SEM), the samples with membranes were mounted on carbon tape, and then Pelco® colloidal silver liquid (Ted Pella Inc.) was introduced onto the bottom part of the PDMS to secure and enhance the conductivity of the PDMS. Once dried, the samples were sputter-coated with carbon and examined by SEM (Tescan XEIA3) at 1 kV.

**Fig. 4:**
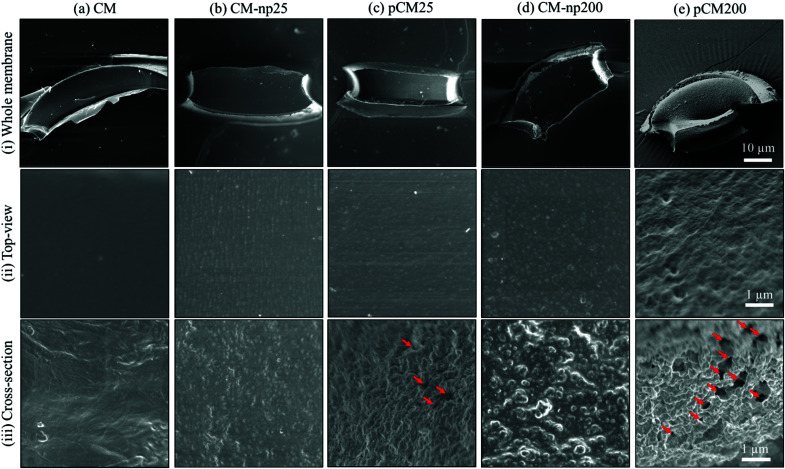
Scanning electron microscopy (SEM) micrographs of (a) CM, (b) CM-np25, (c) pCM25, (d) CM-np200, and (e) pCM200: (i) whole membranes recovered from PDMS microchannels; (ii) membrane morphology of the top surfaces beneath PDMS, and (iii) cross-sectional views of the membranes. Note: Red arrows indicate interconnected pores.

To observe the cross-sections of the membrane porosity (Fig. 4(iii)), another set of membranes with extended length up to 400 μm was flow-assembled in microchannels instead of in apertures in two-PDMS-layer devices. In this case, one output of the device was blocked with a metal plug during the membrane fabrication process, so that the chitosan solution was forced to pass through the aperture to interact with the alginate solution directly and form an elongated membrane in the other output channel instead of within the aperture. Thin layers of the devices about one mm thick containing the cross-sectional membranes were dissected using a sharp razor blade. The thin PDMS layers were mounted on carbon tape and observed by SEM.

### Membrane semi-permeability

**Fig. 5:**
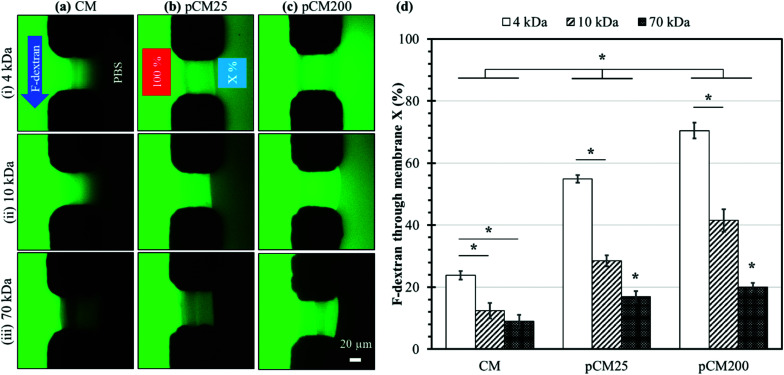
Membrane permeability characterized with F-dextran. Representative fluorescence images of the (a) CM, (b) pCM25, and (c) pCM200 membranes under the permeability test with F-dextran molecules of different size: (i) 4 kDa, (ii) 10 kDa, and (iii) 70 kDa. (d) The percentage of F-dextran that passed through the membrane; error bars represent the standard deviations of 5 measurements. Note: the asterisk (*) indicates *p* < 0.05 between groups using two-way ANOVA with *post hoc* pairwise comparisons.

The membrane semi-permeability was examined in the single-aperture devices using FITC and F-dextran with different molecular weights of 4, 10 and 70 kDa. Test solutions containing 0.1% w/v FITC or F-dextran were continuously introduced through the left microchannel at a flow rate of 1 μL min^–1^, while the right microchannel was filled with PBS in static conditions under stopped flow (Fig. 5(a)-(i)). The semi-permeability of the membranes was quantified by the percentage of F-dextran diffusing across the membranes (*X*%). The average fluorescence of a 50 × 10 μm^2^ rectangle in the left and right channels adjacent to the membrane, abbreviated as left fluorescence and right fluorescence, correspondingly, was measured using ImageJ. The average left fluorescence was set as 100% and the average right fluorescence *X*% was calculated using the following equation:
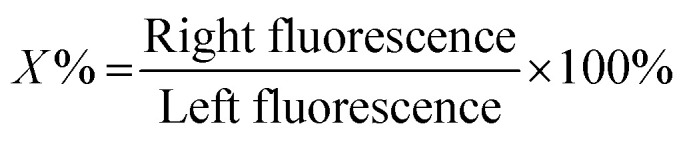


### Membrane crystallization

In a previous study, we reported prominent birefringence signal indicating the crystallization of CM in a microfluidic device using calibrated quantitative polarized light microscopy (qPLM) (Meiji Techno America, MT9930).^4^ Here, the optical retardance *Γ*, a parameter directly proportional to birefringence, was obtained according to previously described techniques^27^,^28^ to examine the impact of GA and DMSO on the crystallization or molecular microstructure of the membranes. Briefly, a single aperture device containing the sample was placed on the qPLM stage. Next, the sample was rotated on the stage until the birefringence signal of the central region of the membrane of interest was the brightest, under crossed polarizers. The sample stage was then locked, with a quarter waveplate inserted below the analyzer, and the analyzer was rotated counterclockwise in 1° steps until all portions of the membrane passed through a minimum pixel value. Images were taken at each angle as the analyzer was turned. The optical retardance was determined from the birefringence signal for each pixel of the images by fitting the pixel signal *versus* analyzer angle to a second-order polynomial, determining the minimum and then generating an optical retardance map of the membrane of interest. The CM, pCM25, and pCM200 that went through the same treatments with GA and then DMSO were characterized by qPLM before and after each step. Three optical retardance maps representing the original, GA-treated, and DMSO-treated membranes were obtained from each sample and the changes in optical retardance in relation to the crystallization of the samples after each treatment were quantified using ImageJ (NIH, USA).

### Parallel membrane arrays for static gradient generation

For the gradient generation of FITC and macromolecules, we employed a microfluidic network comprising three channels as shown in Fig. 3. During gradient generation, the middle channel was filled with PBS and a static middle channel was created by blocking the input port with a catheter plug and connecting the output to a short segment of PBS-filled PTFE tubing (∼1 cm) and finally completely sealing with a small piece of parafilm. The experiments were conducted by continuously introducing 0.1% w/v FITC or F-dextran, representing macromolecules, solution and PBS buffer solution into the source and sink side channels, respectively, at a flow rate of 1 μL min^–1^. The gradients generated in the middle channel were monitored and the plot profiles at 20, 60, 300, 600 and 1800 seconds were obtained using ImageJ and plotted in Fig. 7. All experiments were conducted at room temperature (21 °C).

### Microscopy and statistics

All the images are taken with either a Nikon TS100 or a Ludesco EXI-310 inverted fluorescent microscope with FITC filters. In the permeability tests and gradient generation experiments, fluorescence images were taken every 20 seconds for the first five minutes, then every five minutes for up to 30 minutes. The light shutter was closed when images were not taken to minimize photobleaching. The SEM images were taken with a Tescan XEIA3 SEM machine at the Maryland NanoCenter and its AIMLab. Images were processed using ImageJ (NIH, MD). The error bars in Fig. 5(d) and Fig. S1 (ESI†) represent standard deviations of five measurements. Two-factor ANOVA and *post hoc* pairwise comparisons were performed to compare between groups using SigmaPlot 12.5 with the level of significance being 0.05.

## Results and discussion

### Porous membrane fabrication

The porosity of a membrane is an important attribute to its semi-permeability as well as selectivity. The current study aims to tune the porosity of the biofabricated CM in microfluidics as shown in Fig. 2(a) to enhance their biological applications. By directly co-assembling CM with polystyrene nanoparticles followed by crosslinking and removing the nanoparticles as schematically depicted in Fig. 2(b), pCM were successfully synthesized.

The micrographs in Fig. 2(c) reveal the fabrication steps and the changes at the microscale of pCM200 through each step. First, the air bubble trapped inside the aperture (Fig. 2(c)-(i)), due to the hydrophobicity of the PDMS surfaces, was vacuumed out using an add-on vacuum chamber in the same way as in the biofabrication of pure CM. The complete dissipation of the air bubble allowed the positively charged chitosan solution with polystyrene of 200 nm in diameter and the negatively charged alginate solution to come into contact, instantaneously forming a PECM. Then the pumps were restarted for CM-np200 to be assembled layer-by-layer on the PECM through the diffusion of hydroxyl ions from the alginate side to the chitosan side. Once the desired membrane thickness (around 20–30 μm) was achieved within 2–3 minutes, the add-on vacuum device was removed, the flows were stopped, and the microchannels were rinsed with PBS at a flow rate of 10–15 μL min^–1^. The *in situ* fabricated CM-np200 as shown in Fig. 2(c)-(ii) was robust, freestanding, and semi-permeable like the pure CM. CM-np200 was uniformly packed with tiny polystyrene beads, making it appear textured and less transparent in brightfield microscopy as compared to the pure CM in Fig. 2(a)-(v).

Prior to the removal of the polystyrene nanoparticles, CM-np200 was crosslinked with GA. GA is a well-known crosslinking agent of chitosan to improve the physiochemical properties of chitosan-based hydrogels.^29^–^31^ To allow the complete crosslinking process, GA was left to interact with CM-np200 for one hour, and then the microchannels were rinsed with PBS. Fig. 2(c)-(iii) shows the morphology of CM-np200 after GA crosslinking, in which no significant change was observed. Next, DMSO was used as a solvent to dissolve the nanoparticles. DMSO is a low toxicity solvent, which can dissolve a wide range of polar and nonpolar substances including polystyrene, whilst it has minimal swelling effects on PDMS.^32^,^33^ This allows the solution to stay in the PDMS device for an adequate amount of time (two hours) to dissolve the polystyrene nanoparticles while avoiding effects on the device microchannels and unexpected leakage. Fig. 2(c)-(iv) shows the microscale morphology of pCM200, which has changed significantly in that the membrane became less textured and more transparent under transmission light. This indicated that the nanoparticles that blocked the transmission light had been removed, leaving the porous structure within pCM200.

Similarly, polystyrene nanoparticles of 25 nm in diameter were co-assembled with CM in flows to form CM-np25, followed by nanoparticle removal to form pCM25.

### Nanoscale morphology of the membranes

Next, SEM was employed to characterize the differences in nanoscale morphology of CM, CM-np and pCM. The top row of the SEM photographs in Fig. 4-(i) shows the whole CM, CM-np, and pCM membranes obtained from single apertures in PDMS devices, which were about 60 × 20 × 40 μm in length × wide × depth. Fig. 4(ii) shows that the CM (Fig. 4(a)) had relatively smooth surfaces, while the CM-np (Fig. 4(b and d)) possessed significantly rougher surfaces. Cross-sectional views of the membranes in Fig. 4(iii) were obtained from membranes with extended length assembled in microchannels instead of within apertures in two-PDMS-layer devices as described in the Experimental section, to facilitate obtaining cross-sections. It was noticed that when the CM was co-assembled with nanoparticles, a uniform deposition of nanoparticles was observed on the surface of (Fig. 4(ii)) and inside (Fig. 4(iii)) the membranes regardless of the nanoparticle size. Next, Fig. 4(c, e)-(ii, iii) show that the number of nanoparticles on the pCM surface was considerably reduced, while the cross-sections of the pCM were highly porous after the removal of nanoparticles using DMSO. Notably, Fig. 4(e)-(iii) shows that pCM200 possessed an interconnected-porous structure with pore sizes of 200–400 nm, which would allow the transport of macromolecules.

### Semi-permeability of the membranes

Many applications with membrane platforms are based on membrane semi-permeability, which allows mass transport and creates selective barriers for certain molecules or ions. As observed in the SEM micrographs, the removal of nanoparticles noticeably altered the nanostructure of the pCM, which might also influence their semi-permeability. The membrane permeability was investigated using F-dextran macromolecules of different sizes as described in Materials and methods. The thickness of the CM and pCM was controlled to be similar to ensure that the membrane thickness is not a possible factor leading to differences in permeability. The size of F-dextran of different molecular weight was calculated using the following equation:*R*_min_ = 0.066*M*^1/3^where *M* is the molecular weight (Da), and *R*_min_ is the minimal hydrodynamic radius (nanometers).^34^ Practically, the size of a given mass of protein might vary in relation to the protein physiochemical properties of that protein as dissolved in solvent.^16^ Here, we employed the hydrodynamic radius estimator from Zetasizer software (Malvern Panalytical Inc., USA) to estimate the hydrodynamic radius of F-dextran of different molecular weight (4 kDa, 10 kDa, and 70 kDa). The estimated results in Table S1 (ESI†) show that the higher the molecular weight, the larger the size of dextran.

Fig. 5(a–c) show representative fluorescence images of the corresponding CM, pCM25, and pCM200 under the permeability test with F-dextran molecules of various size, whilst Fig. 5(d) shows the corresponding percentages of F-dextran that passed through CM, pCM25, and pCM200 after the first ten minutes. The fluorescence images in Fig. 5(a) qualitatively show that (1) the minimum fluorescence signal was observed on the PBS side of the CM; and (2) similar levels of fluorescence signal were observed within the CM for the cases of 4 and 10 kDa dextran, while that was lower for the case of 70 kDa. The fluorescence images in Fig. 5(b) show that (1) obvious fluorescence signal was observed on the PBS side of pCM25 for the cases of 4 and 10 kDa dextran, while that was minimum for the case of 70 kDa; and, interestingly, (2) the maximum fluorescence signal was observed within pCM25 for the case of 10 kDa as compared to that for the 4 and 70 kDa cases. The fluorescence images in Fig. 5(c) show a similar tendency to those in Fig. 5(b) except that the fluorescence signal on the PBS side was much brighter as compared to that in Fig. 5(b). The fact that the maximum fluorescence within the membranes was observed for the cases of 10 kDa for pCM25 and pCM200 instead of the other cases is probably due to two possible reasons. First, some sort of fluorescence quenching might have happened when excessive fluorescent dye accumulated within the membranes. This was more obvious in Fig. 7(a), which will be discussed further. Second, more 10 kDa molecules might have accumulated inside pCM25 and pCM200 than 4 kDa molecules, while the 4 kDa molecules could have diffused through the porous membranes more easily. Figuring out the exact reason is worth further investigation in the future. Overall, the larger the molecular weight, the smaller the number of molecules that passed through the membrane, and the CM allowed a significantly smaller number of fluorescent molecules to pass through as compared to the pCM.

The permeability was further quantified with the percentages of F-dextran that passed through the CM, pCM25, and pCM200 based on the spatial fluorescence intensity of the obtained micrographs. The results in Fig. 5(d) show that the CM allowed a relatively small percentage of 23.8 ± 1.4, 12.4 ± 2.4 and 9.0 ± 2.1 of the corresponding 4, 10 and 70 kDa dextran to pass through. Meanwhile, these numbers for pCM25 were almost doubled to 54.8 ± 1.2, 28.5 ± 1.8 and 16.9 ± 1.8 for 4, 10 and 70 kDa dextran, respectively. Lastly, pCM200 with the largest pore size allowed up to 70.5 ± 2.5, 41.5 ± 3.6 and 20.0 ± 1.3 percent of 4, 10 and 70 kDa dextran to pass through, respectively. For the control experiments, the permeability of the CM-np25 and CM-np200 membranes before the embedded nanoparticles were dissolved was also examined and showed comparable results to the CM cases. Specifically, CM-np25 allowed 24.4 ± 1.5, 15.3 ± 1.0, and 9.8 ± 0.7 percent of the corresponding 4, 10, and 70 kDa dextran to pass through, while these numbers for CM-np200 were 24.0 ± 1.1, 14.1 ± 1.7, and 12.8 ± 1.1, respectively (Fig. S1, ESI†). These control experiments indicate that the co-assembly of the CM with nanoparticles did not significantly alter the permeability of the membranes.

Furthermore, we confirmed with a new set of control experiments that the GA crosslinking step was crucial in maintaining the porous structure of the pCM after removing the polystyrene nanoparticles. For this, the co-assembled CM-np were directly treated with DMSO for nanoparticle removal without GA crosslinking, resulting in 
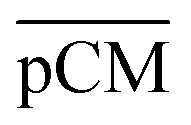
. Fig. S1 (ESI†) also shows the percentages of F-dextran through 
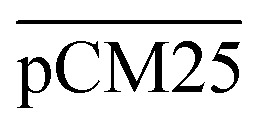
 and 
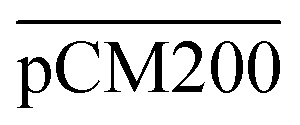
. The percentages of F-dextran of 4, 10, and 70 kDa that passed through 
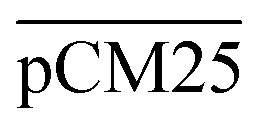
 were 23.1 ± 1.5, 13.5 ± 0.7, and 10.8 ± 1.0, whilst the figures for 
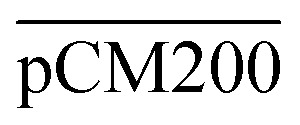
 were 22.7 ± 1.7, 13.3 ± 1.0, and 13.1 ± 0.8, respectively. Overall, these control experiments showed that the permeability of 
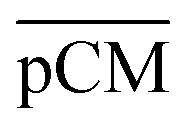
 remained nearly similar to those of the CM and CM-np and was significantly less than that of the pCM. The crosslinking between the aldehyde groups of GA and amine groups of deprotonated chitosan to form imine groups had improved the assembled membranes’ mechanical and structural robustness.^31^ Without crosslinking, the free and elastic amine groups within CM-np tended to collapse after the polystyrene nanoparticles were removed, thus disabling the transport of macromolecules.

### Membrane crystallization

**Fig. 6:**
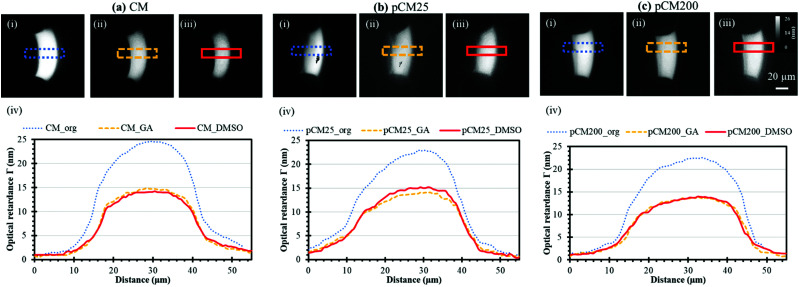
Membrane birefringence indicating crystalline alignment characterized by quantitative polarized light microscopy. Birefringence images of (a) CM, (b) pCM25, and (c) pCM200: (i) original membranes; (ii) membranes treated with GA, and (iii) membranes treated with DMSO, and (iv) the gray scale plots of the corresponding membranes. Note: membranes were named as follows: membrane Type_Treatment abbreviation.

The crystallization of the CM and pCM can be revealed through birefringence, which has two components: intrinsic and form,^35^ and the relative contributions of these two kinds of birefringence to the signal from chitosan are not clear yet. Intrinsic birefringence is related to the co-orientation of anisotropic bond polarizabilities within and between molecules.^36^ We hypothesize that bulk chitosan membrane birefringence is related to its semi-crystalline nature, but it could also be influenced by solvent effects and other interactions. Therefore, the crystallization or molecular microstructure of the CM and pCM revealed through birefringence is another important parameter to be examined. The optical retardance *Γ*, a parameter correlated to birefringence, was determined from the optical retardance map of the membrane as described in the Experimental section. A higher optical retardance signal indicates higher crystalline order within the membrane.^4^,^37^ Fig. 6(a)-(i–iii) show the optical retardance maps of the original CM, CM treated with GA, and CM treated with DMSO, while the profiles of optical retardance across the corresponding membranes are plotted in Fig. 6(a)-(iv). The results show that the net optical retardance was around 24.3 ± 1.5 nm for the original CM but dropped to 14.7 ± 0.6 nm, or a 40% decrease, after GA treatment, and remained almost the same as those with GA crosslinking after the additional DMSO treatment (14.3 ± 0.9 nm). Similar trends were observed for pCM25 and pCM200 as shown in Fig. 6(b and c). The slightly lower optical retardance of pCM25_org (22.9 ± 1.2 nm) and pCM200_org (22.6 ± 1.0 nm) as compared to CM_org indicates that the co-assembly of nanoparticles did not noticeably alter the membranes’ crystallization.

These results suggest that (1) GA crosslinking had altered the microstructure of the highly-aligned flow-assembled CM, which is similar to our recent report,^9^ while (2) DMSO treatment had little or no effect on the GA-crosslinked CM structure, which is new in this study. This is in agreement with the literature, in which no study has reported the interaction of chitosan with organic solvent such as DMSO.^38^ The lack of change in optical retardance with DMSO indicates that higher FITC-dextran transport across membranes is not likely due to altered chitosan packing and organization. This also indicates that the differences in mass transport of various size F-dextran were likely caused by the interconnected pores but not the membrane crystallization.

### Static gradient generation with parallel membrane arrays

After the porous structure of the pCM had been observed and its significantly enhanced semi-permeability to macromolecules as compared to the CM had been confirmed, we explored the generation of a steady gradient of small and macro-molecules in three-channel devices composed of two parallel pCM arrays (pCM gradient generator) and compared the results to those composed of two parallel CM arrays (CM gradient generator).

**Fig. 7:**
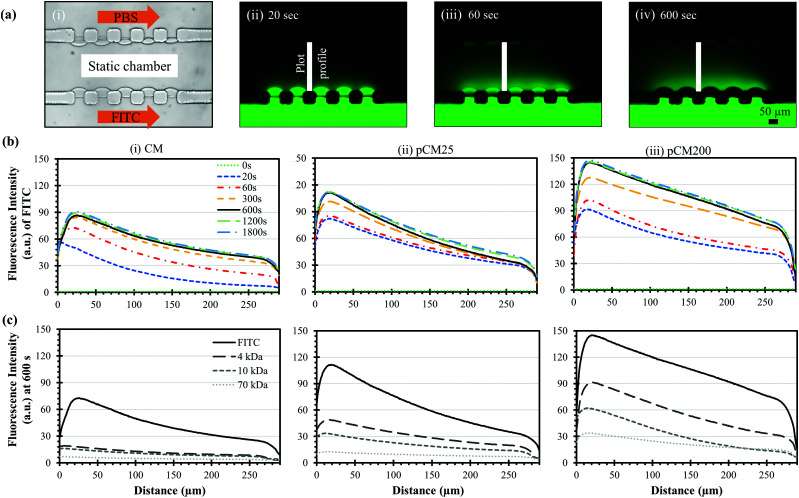
Static gradients of FITC and macromolecules across parallel membranes in three-channel devices. (a)-(i) Experimental setup to generate static gradients in the middle channel of a device with parallel CM and the time evolving images of static FITC gradients at (ii) 20, (iii) 60 and (iv) 600 seconds; and (b) the time evolving FITC gradient profiles as in (a) in the middle channels with parallel (i) CM, (ii) pCM25 and (iii) pCM200. (c) The static gradient profiles of molecules with different sizes (FITC and F-dextran of 4, 10 and 70 kDa) at 600 seconds in three-channel devices of different parallel membranes.

Fig. 7(a)-(i) shows the experimental setup, in which FITC or F-dextran solution of 1 mg mL^–1^ was introduced into the source channel while PBS solution was introduced into the sink channel, both under continuous flow at a flow rate of 1 μL min^–1^. The middle channel was filled with PBS buffer and maintained in static conditions as described in the Materials and methods section. Fig. 7(a)-(ii–iv) show representative fluorescence images of the established FITC static gradients in the middle channel of a CM gradient generator device at 20, 60 and 600 seconds. Notably, the gradient of the fluorescence signal in the static middle channel was quickly established within minutes. However, the fluorescence signal within the membranes became darker in Fig. 7(a)-(iii) and completely disappear in Fig. 7(a)-(iv), although presumably more FITC molecules had accumulated in the membranes. We speculate that this is due to some sort of fluorescence quenching resulting from excessive fluorescent dye accumulation within the membranes as previously discussed in Fig. 5(a–c). On the other hand, quenching was not observed within the middle channel up to 30 minutes as shown in Fig. 7(b). Further investigation is needed to figure out the fluorescence quenching within chitosan membranes.

Fig. 7(b)-(i) shows the evolving fluorescence gradients of FITC over time within the parallel CM corresponding to the indicated plot lines in Fig. 7(a)-(ii–iv). Overall, there was a nearly linear static gradient established within 300 seconds, and that linear static gradient was well-maintained after that as the corresponding plot profiles mostly overlap. The gradient profiles were slightly nonlinear near the sink side after the static gradient was established since the plot profiles were taken from the middle edges of the PDMS pillars instead of from the membrane surfaces. Fig. 7(b)-(ii and iii) show similar trends within the pCM gradient generators where the FITC gradients were quickly responsive and remained steady over time. Significantly, the FITC gradients generated in a pCM gradient generator was noticeably steeper as compared to those in a CM gradient generator, and the fluorescence signal in the static gradients representing the molecule quantity increased significantly with the increase of membrane porosity from the CM to pCM25 to pCM200.

Fig. 7(c) shows the established static gradients of FITC and F-dextran of 4, 10 and 70 kDa at 600 seconds in the CM, pCM25 and pCM200 gradient generators. In the case of the CM gradient generator in Fig. 7(c)-(i), since the molecular weight of FITC is only about 0.4 kDa, FITC could effortlessly penetrate through the parallel CM and generated a significantly steep gradient. However, only a moderate portion of F-dextran could pass through the CM, creating relatively flat gradients in the static middle channel as compared to FITC. As the molecular weight of F-dextran increased, the gradient established across the CM became flatter. In the case of the pCM gradient generators in Fig. 7(c)-(ii and iii), not only were the FITC gradients steeper as discussed previously, but also the F-dextran quantity was significantly increased as compared to that in a CM gradient generator. Similarly, the F-dextran gradients in the pCM gradient generators became flatter with the increase of the molecular weight of F-dextran. Significantly, the gradients of macromolecules increased and became steeper with the higher membrane porosity from pCM25 in Fig. 7(c)-(ii) to pCM200 in Fig. 7(c)-(iii).

**Fig. 8:**
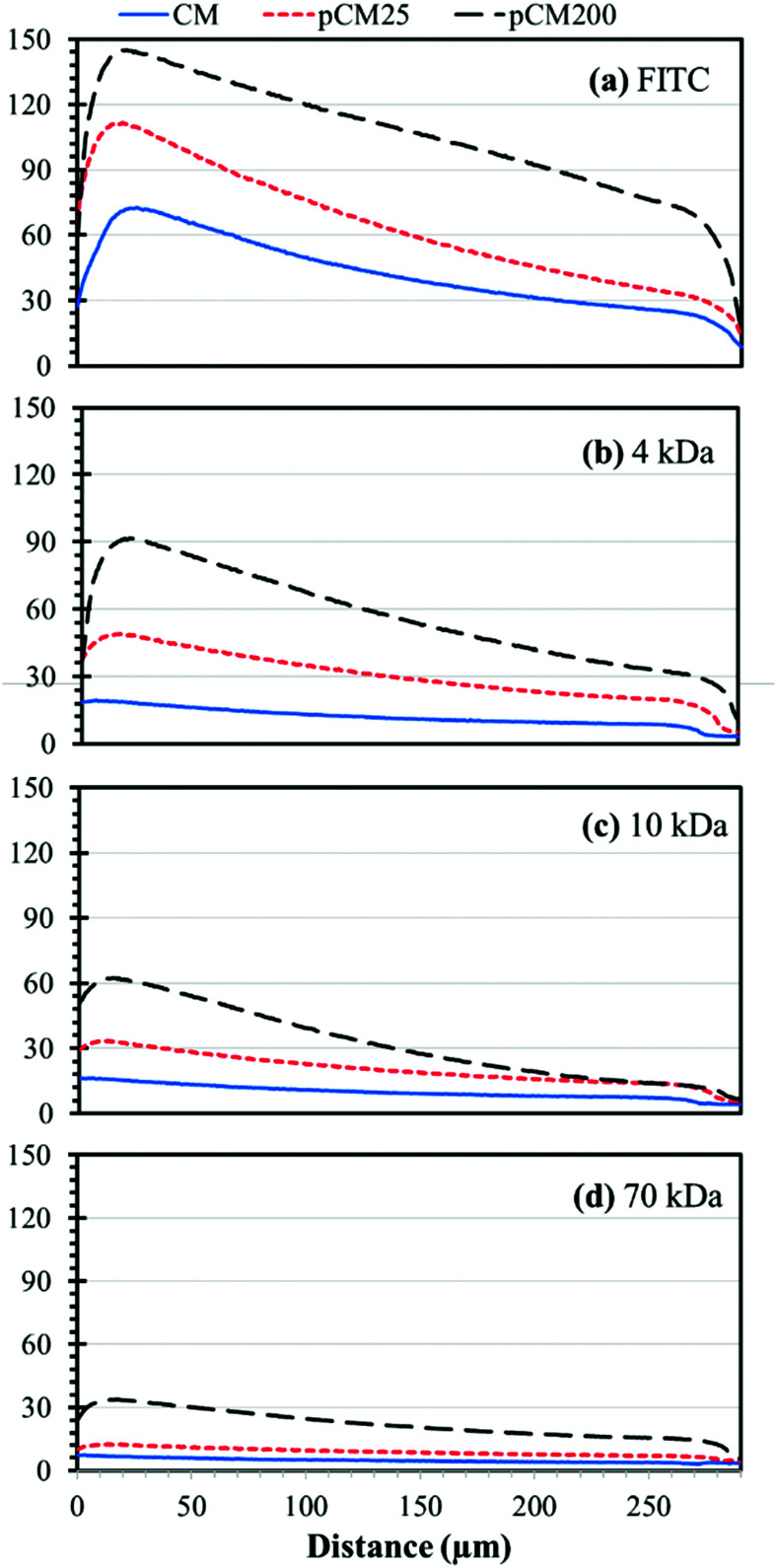
The static gradient profiles of different molecules at 600 seconds in the middle channels of devices with parallel CM, pCM25 or pCM200: (a) FITC, (b) 4 kDa dextran, (c) 10 kDa dextran, and (d) 70 kDa dextran.

For better comparison and from an application point of view, we re-plotted the static gradient profiles in the CM, pCM25 and pCM200 gradient generators into groups of fluorescent molecules, representing biological molecules of different sizes, as shown in Fig. 8. For FITC, representing small molecules, the gradient levels were significantly increased with the increase of the membrane porosity, while the steepness or slopes of the gradient profiles were only moderately increased with enhanced membrane porosity. For 4 kDa F-dextran, representing peptides, both the gradient levels and steepness of the gradient profiles were significantly increased with enhanced membrane porosity. In fact, the gradient of 4 kDa F-dextran was almost flat in the CM gradient generator, suggesting that the pCM gradient generators but not the CM device should be used for medium molecular weight peptides. For 10 kDa F-dextran, representing some small proteins or protein monomers, the major noticeable change with the enhanced membrane porosity was the increase of the steepness of the gradient profiles: the more porous the membranes, the sharper the gradients. For 70 kDa F-dextran, only the pCM200 device could generate moderately steep gradients. In this case, a further increase of the membrane porosity with either a higher density of nanoparticles as a template or bigger size particles should be considered.

To summarize, microfluidics offers many benefits towards concentration gradient generation for biochemical and cellular studies.^21^,^39^ In particular, microfluidic-based platforms can generate predictable, reproducible and quantitative gradients with low sample consumption and quick response time.^21^,^40^,^41^ Our static gradient generator composed of parallel and semipermeable biopolymer membranes in a single layer PDMS device attains numerous characteristics of a good gradient generation platform. The biofabrication process is simple and mild, and yet is able to produce robust membranes that can withhold up to one atmosphere pressure^1^,^25^ and generate static gradients of small molecules over time within a few minutes and maintained steadily over time (Fig. 7(b)-(i)). To improve the applicability of our gradient generator of small, medium and macro-biomolecules for diverse biochemical and cellular studies, here we demonstrated the tuning of the membrane porosity using polystyrene nanoparticles as a sacrificial template. By directly co-assembling CM with polystyrene nanoparticles followed by crosslinking and removing nanoparticles as schematically depicted in Fig. 2(b), pCM were successfully synthesized. The porous structure and significantly enhanced permeability to macromolecules of the synthesized pCM was demonstrated as compared to the CM (Fig. 4 and 5). The crystallization data indicated that GA crosslinking had altered the microstructure of the highly aligned flow-assembled CM, while DMSO treatment had little or no effect on the GA-crosslinked CM structure (Fig. 6). Similar trends happen among pCM that underwent the same treatment. Permeability of macromolecules through the pCM was demonstrated and compared with that through the original CM (Fig. 5), confirming the capability to actively tune the membrane porosity according to application needs. The tuning process increased the pore size of the membranes and, therefore, enabled a wide range of macromolecule concentration gradients to be generated through the pCM as compared with that through the CM (Fig. 8(b–d)), which could capture more sensitive responses correlating to the concentration gradients. Further tuning and optimization could be done based on application needs by following the reported procedure.

## Conclusions

In this study, we have successfully customized the porosity of CM in microfluidics using co-assembled nanoparticles as templates. The biofabrication process is facile, mild and reliable with the assistance of a steering air bubble technique, the biofabricated membranes are robust, and the membrane growth is spatially and temporally controllable. We demonstrated here that the biofabricated pCM were highly porous with interconnected pores, which significantly enhanced their permeability to biomacromolecules such as peptides and proteins. Steep gradients of different size fluorescently labelled macromolecules were generated quickly and yet were steady over time through the biofabricated pCM as compared to the CM. These pCM are promising and should be further investigated as a promising platform for biomolecular and cellular studies.

## Conflicts of interest

There are no conflicts to declare.

## Supplementary Material

Supplementary information
