# Supplementary material for: Tuning the porosity of biofabricated chitosan membranes in microfluidics with co-assembled nanoparticles as templates
Source: Mater Adv. 2020 Mar 11;1(1):34–44. doi: 10.1039/d0ma00073f (PMC7518516; doi:10.1039/d0ma00073f)
Supplement: Supplementary file 1 [file MA-001-D0MA00073F-s001.pdf]

Electronic Supplementary Material (ESI) for Materials Advances.
This journal is © The Royal Society of Chemistry 2020

Supplement

Table S1. The estimated hydrodynamic radius for F-dextran of different mass obtain using the hydrodynamic radius estimator of Zetasizer software.

F-Dextran (kDa)	4	10	70
**Estimated hydrodynamic radius (nm)**	1.08 – 1.71	1.59 – 2.41	3.66 – 7.04

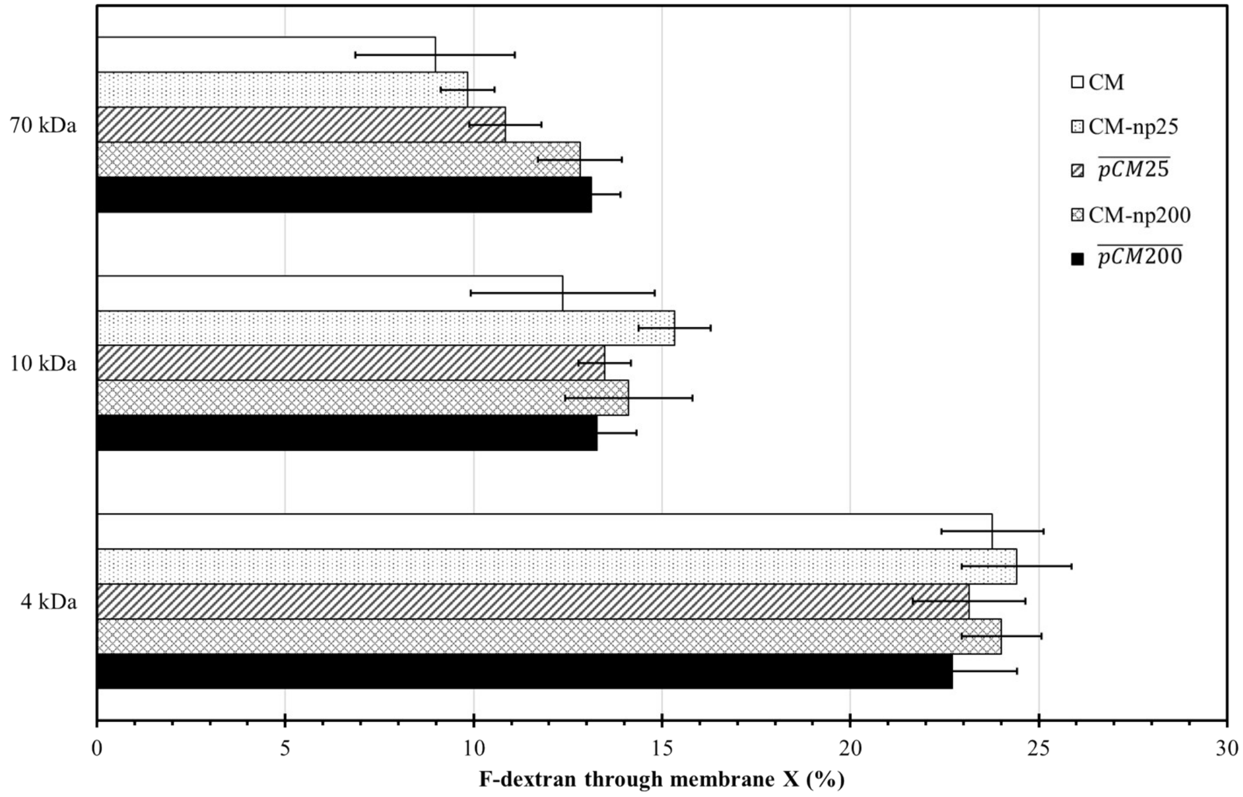

Fig S1. The percentage of F-dextran passed through CM, CM-np25, CM-np200, $pC\bar{M}25$, and $pC\bar{M}200$ as controls; error bars represent the standard deviations of 5 measurements. *Note:* $pC\bar{M}25$ and $pC\bar{M}200$ are membranes treated directly with DMSO without crosslinking.
